# A Case Report of a Rare 46,XX/47,XXY Mosaicism With Ovotesticular Disorder of Sex Development and a Literature Review

**DOI:** 10.7759/cureus.65856

**Published:** 2024-07-31

**Authors:** Mohamed Hssaini, Ghita Bourkadi, Mohamed Ahakoud, Laila Bouguenouch, Sanae Abourazzak, Hicham Bekkari, Amina Ameli

**Affiliations:** 1 Medical Genetics and Oncogenetics Laboratory, Hassan II University Hospital, Fez, MAR; 2 Laboratory of Biotechnology, Environment, Food, and Health, Faculty of Sciences Dhar El Mahraz, Sidi Mohamed Ben Abdellah University, Fez, MAR; 3 Department of Endocrinology, Diabetology, and Nutrition, Hassan II University Hospital, Fez, MAR; 4 Department of Pediatric Endocrinology, Hassan II University Hospital, Fez, MAR

**Keywords:** morocco, atypical genitalia, klinefelter syndrome mosaicism, ovotesticular disorder of sex development, 46 xx/47 xxy mosaicism

## Abstract

Klinefelter syndrome (KS) is a common chromosomal abnormality in males, usually presenting as a 47,XXY karyotype and often underdiagnosed. Rarely, KS occurs as mosaic 46,XX/47,XXY. At the same time, ovotesticular disorder of sex development (OT-DSD) is also a rare condition in which both ovarian and testicular structures are present in the same individual, often associated with a 46,XX karyotype. The combination of mosaic 46,XX/47,XXY with OT-DSD is scarce. Herein, we report a new case of a six-month-old infant with unilateral OT-DSD and a 46,XX/47,XXY mosaic karyotype who presented with atypical genitalia at birth. On examination, the external genitalia showed asymmetry of the labioscrotal folds, an empty right fold, a 2.5 cm phallic structure, and a perineal urethral meatus. Imaging studies revealed a uterus and a vaginal cavity, as well as an ovotestis on the left side and an ovarian remnant on the right side. An unexpected increase in testosterone level was observed. Cytogenetics analysis confirmed a mosaic karyotype with 54% of 46,XX and 46% 47,XXY cells. Molecular genetic analysis revealed no mutations in the genes involved in gonadal development. These findings are discussed and the clinical characteristics of the reported cases of 46,XX/47,XXY with OT-DSD are summarized. In conclusion, atypical genitalia leads to the early diagnosis of the rare 46,XX/47,XXY mosaicism with OT-DSD. Mosaicism should be considered in all cryptorchidism cases. Persistent Müllerian structures were common, and the nearly male phenotype of the external genitalia led parents to prefer the male sex of rearing.

## Introduction

Klinefelter syndrome (KS) is a common chromosomal abnormality in humans, affecting approximately one in 600 live-born males [1]. Often underdiagnosed until adulthood due to mild clinical symptoms, KS is defined by various features such as testicular abnormalities, gynecomastia, tall stature, sparse body hair, androgen deficiency, hypergonadotropic hypogonadism, and normal to slightly decreased verbal intelligence [2]. While 80% of KS cases have a 47,XXY karyotype, the remaining 20% involve other chromosomal abnormalities or mosaicism [3]. A mosaic 46,XX/47,XXY karyotype is very rare. On the other hand, ovotesticular disorder of sex development (OT-DSD) is a rare gonadal development disorder, comprising approximately 3-10% of disorder of sex development (DSD) cases [4]. This condition is marked by the simultaneous presence of ovarian and testicular tissues (ovotestis) within the same individual [5]. Furthermore, OT-DSD can be classified into three types: lateral (ovary on one side and testis on the other), bilateral (ovotestis on both sides), or unilateral (ovary or testis on one side and ovotestis on the other).

Unlike KS, overt atypical genitalia is observed in up to 90% of OT-DSD cases [6]. The phenotypic presentation of OT-DSD is highly variable, ranging from typical male to typical female appearances [5,7]. The most commonly observed karyotype in OT-DSD cases is 46,XX, followed by mosaic patterns such as 46,XX/46,XY and 46,XY [7]. Cases involving OT-DSD and mosaicism are more complex and extremely rare.

To date, there are only 12 published cases of 46,XX/47,XXY mosaicism associated with lateral (five cases) [8-12], unilateral (six cases) [5,13-17], and bilateral (one case) OT-DSD [18]. Our patient represents the seventh reported case of unilateral OT-DSD.

In this report, we present the description of a new case of a six-month-old infant with a rare subset of unilateral OT-DSD associated with a rare variant of mosaic KS, 46,XX/47,XXY. Molecular studies were performed on several genes to uncover potential hidden genetic causes not evident through karyotyping and fluorescent in situ hybridization (FISH) alone. Additionally, we summarized the clinical features and gonadal characteristics of similar published cases.

## Case presentation

The patient was a six-month-old infant, the youngest of three siblings, born to non-consanguineous parents. The mother, aged 35 years, had a history of three unexplored miscarriages. The pregnancy was well monitored and carried to term. Delivery was by cesarean section, with a birth weight of 3300 grams.

**Figure 1:**
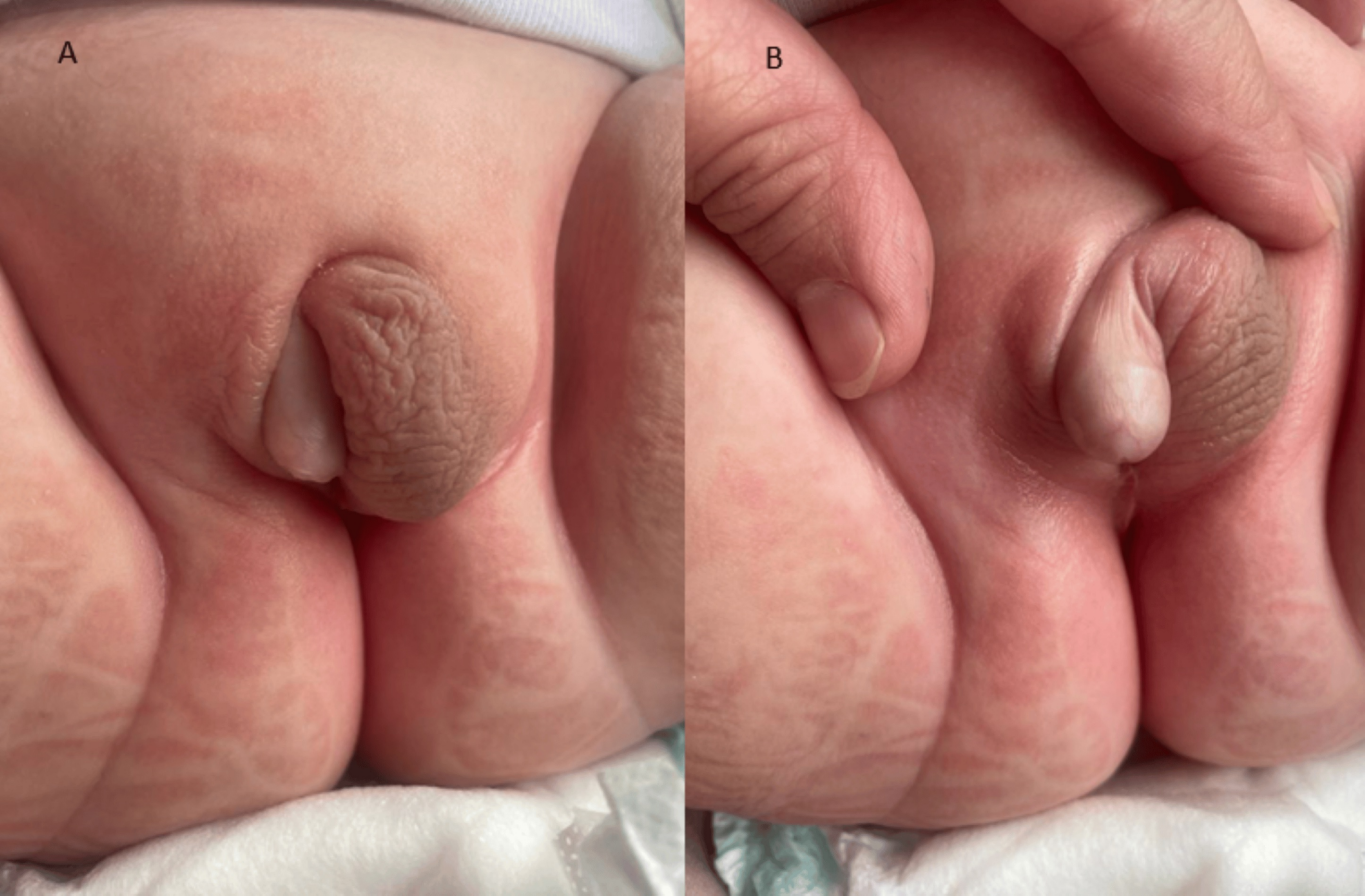
Appearance of the external genitalia. A: Asymmetry of the labioscrotal folds. B: Presence of phallic structure.

The infant was admitted to our clinical department on the third day of life for evaluation of atypical genitalia. Clinical examination revealed a reactive and tonic neonate without signs of dehydration. Examination of the external genitalia showed an asymmetry of the labioscrotal folds, with a slightly pigmented striated appearance. The right labioscrotal fold was empty, while the left contained a 1 x 0.5 cm testis. There was also a 2.5 cm phallic structure and a perineal urethral meatus (Figure 1). The remainder of the physical examination was apparently normal.

Radiological evaluation with pelvic and scrotal ultrasound showed a vertically elongated structure behind the bladder, suggestive of a uterus (27 x 13 mm), and a homogenous, well-defined nodular formation in the left external genitalia resembling a testis (13.6 x 6 mm).

**Figure 2:**
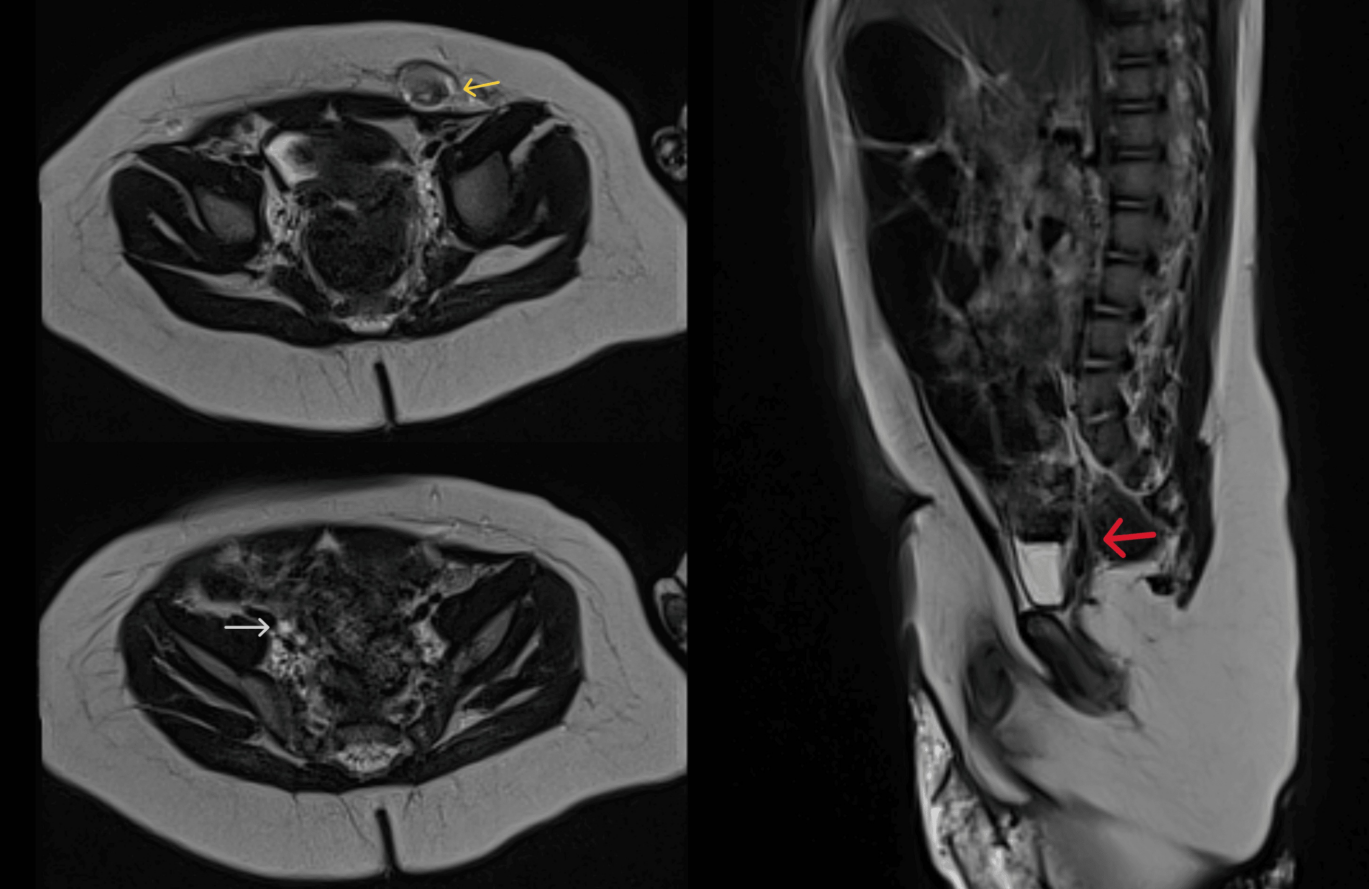
Magnetic resonance imaging highlighting the presence of the uterus (red arrow), left testis (yellow arrow), and right ovarian remnant (white arrow).

A pelvic MRI revealed a right-sided retrovesical uterus (15 mm) with a 2 mm endometrium, a 7 mm vaginal cavity, and small ovarian remnants (3 mm right, 2 mm left). On the left side, a high inguinal structure compatible with a testis (8 mm) with minimal hydrocele was noted, as well as a hypertrophied clitoris. The urethra was the female type, and no prostate or right testis was observed (Figure 2).

Routine laboratory tests, including serum electrolytes and renal function, were unremarkable. The hormonal evaluation at seven days of age revealed slightly elevated levels of testosterone (T) at 0.92 ng/mL, anti-Müllerian hormone (AMH) at 14.77 µg/L, and luteinizing hormone (LH) at 4.88 mIU/mL. Other hormone levels were considered age-appropriate: dihydrotestosterone at 0.09 ng/mL, follicle-stimulating hormone (FSH) at 2.19 mIU/mL, adrenocorticotropic hormone (ACTH) at 16.9 pg/mL, and 17-hydroxyprogesterone at 4.5 ng/mL (Table 1).

**Table 1:** Initial hormonal status of the patient.

Hormonal assay	Patient value	Reference value
Testosterone (ng/mL)	0.92	<0.2
Dihydrotestosterone (ng/mL)	0.09	0.05-0.4
Luteinizing hormone (mIU/mL)	4.88	<3.6
Follicle-stimulating hormone (mIU/mL)	2.19	<5
Anti-Müllerian hormone (µg /L)	14.77	1.5-11.8
Adrenocorticotropic hormone (pg/mL)	16.9	4.7-48.8
17-hydroxyprogesterone (ng/mL)	4.5	<2.5

**Figure 3:**
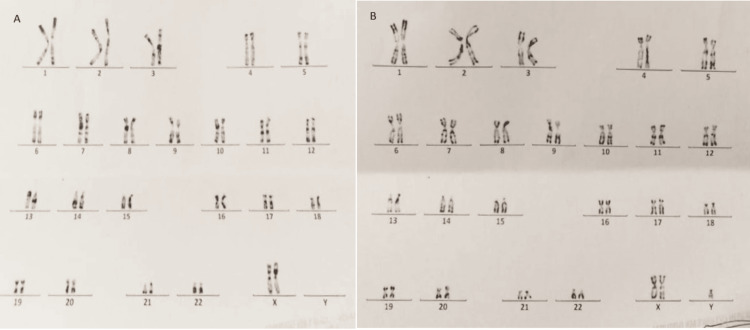
Karyotype of the patient. A: Karyotype showing a 46,XX cell line. B: Karyotype showing a 47,XXY cell line.

Chromosome analysis was performed on peripheral blood lymphocytes using R-HG banding with a resolution of 5-10Mb according to standard procedures. Karyotyping analysis showed mosaicism with two types of cells: a predominant cell line of normal female chromosome 46,XX in 54% of the examined cells (14 cells), and a 47,XXY cell line in 46% of the examined cells (12 cells) (Figure 3). FISH using the probe LSI SRY/CEPX (Vysis, Abbott, Abbott Park, IL) confirmed the presence of SRY and the mosaicism. The parents had normal karyotypes.

Sanger sequencing was performed for the SRY gene. Additionally, we investigated the roles of 49 genes (Appendices) involved in sexual differentiation disorders by sequencing specific regions (coding exons) using the Ion GeneStudio™ S5 System (Life Technologies Corporation, Carlsbad, CA). DNA samples were prepared using the Ion AmpliSeq™ Kit for Chef DL8 and the Ion Chef™ System (Thermo Fisher Scientific, Waltham, MA) according to the manufacturer's protocol. The DNA libraries were quantified using the Ion Library TaqMan™ Quantitation Kit, then templated on Ion 530™ Chips with the Ion Chef™ System and sequenced on the Ion GeneStudio™ S5. The raw data generated were analyzed using both Torrent Suite (Thermo Fisher Scientific) and Ion Reporter (Thermo Fisher Scientific), which together provide a comprehensive bioinformatics workflow. Torrent Suite was used initially for quality control, including the removal of low-quality reads and trimming of adapter sequences. It also facilitated the alignment of reads to the reference genome and the initial variant calling, identifying single nucleotide variants (SNVs), insertions, deletions (indels), and copy number variations (CNVs). Following this, Ion Reporter was utilized for further variant analysis. This includes detailed annotation, where variants were cross-referenced with multiple databases for information on gene function, pathogenicity, and population frequency. Filtering steps were applied to focus on variants of clinical significance, based on quality metrics and known disease associations. The analysis culminated in the generation of detailed reports summarizing the identified variants, their potential impacts, and clinical interpretations, thus providing valuable insights into the genetic factors contributing to sexual differentiation disorders. Yet, this analysis did not detect any mutations in all related genes.

The patient, initially assigned male, is currently under follow-up by a multidisciplinary team. The parents are open to reconsidering the child’s sex based on gonadal function assessment results and future fertility prospects. Surgical interventions and hormonal treatments will be evaluated in light of these factors.

## Discussion

Mosaic 46,XX/47,XXY is a rare variant of KS. Unlike the more common non-mosaic form (47,XXY), which is typically caused by paternal non-disjunction of the sex chromosomes during meiosis [19], the origin of the two cell lines in the 46,XX/47,XXY variant is more complex. In our patient’s case, the most likely explanation is a post-zygotic loss of a Y chromosome from an initial 47,XXY zygote, rather than the loss of an X chromosome, giving rise to the 46,XY cell line. The possibility of parental gonadal mosaicism is an unlikely cause of our patient’s genotype.

The phenotypic heterogeneity and mild clinical signs of KS often lead to late diagnosis until adulthood, resulting in a lack of early intervention and treatment [2]. However, the presence of additional signs of another pathology may allow an earlier detection and more rapid diagnosis. This was observed in patients with associated OT-DSD who present with overt atypical genitalia at birth or during childhood [10,12,15,17]. Furthermore, OT-DSD is a rare condition and even rarer when associated with 46,XX/47,XXY mosaic.

Following an exhaustive literature review, to date, there have been fewer than 12 reported cases of mosaic 46,XX/47,XXY in OT-DSD worldwide [5,8-18]. Among these, only six cases involved unilateral OT-DSD. Our case represents the seventh case of mosaic 46,XX/47,XXY with unilateral OT-DSD and the first to be reported from Morocco.

According to the literature (Table 2), the age of diagnosis for OT-DSD shows two major peaks. The first peak occurs in childhood (4/12 cases), with ages ranging from 27 weeks of gestation to 1.7 years, often due to the presence of atypical genitalia [10,12,15,17]. Only one patient was diagnosed later at 16 years old and had atypical genitalia [13]. As seen in our patient, atypical genitalia was identified immediately after birth, leading to an early diagnosis. The second peak occurs after 12 years of age (8/12 cases), where complaints commonly include scrotal pain with or without hematuria (four cases), followed by gynecomastia, female body habitus, and a scrotal mass in three cases. All pubertal patients were reared as male due to their male phenotypic appearance or behavior, though a complete male phenotype is rare. Only one case was reported for near-complete male external genitalia in a 14.5-year-old boy evaluated initially for hematuria [14]. While female or near-female external genitalia are observed separately in OT-DSD or 46,XX/47,XXY individuals [20,21], none of the reported cases with OT-DSD and 46,XX/47,XXY mosaicism presented with completely or nearly female external genitalia, thus, none were raised as female. Gynecomastia was present in all patients over the age of 12 years. Including our patient, 83.3% (10/12) of all individuals had unilateral or bilateral cryptorchidism [5,10-14,17,18].

**Table 2:** Characteristics of reported cases of patients with rare mosaicism 46,XX/47,XXY and OT-DSD. WK GA: week gestation; OT: ovotestis; O: ovary; T: testis; NA: data not available or not applicable; OT-DSD: ovotesticular disorder of sex development; Testo: testosterone; FSH: follicle-stimulating hormone; LH: luteinizing hormone; ^R^: in the right gonad; ^L^: in the left gonad; ^a^: presence of hypoplastic fallopian tube; +: present; -: absent.

Patient	Age (years)	Reason for admission	External genitalia	Sex of rearing	Gynecomastia	Right gonad	Left gonad	Uterus	Vas deferens	Percentage of 46,XX (%) in peripheral blood	Hormone level			Learning difficulties	References
											Testo	FSH	LH		
1	3 days	Atypical genitalia	Perineal hypospadias, asymmetry of labioscrotal folds, cryptorchidism	Male	-	O	OT	+	-	54	High	Normal	High	NA	This study
2	27 WK GA	Atypical genitalia	Bifid scrotum, hypospadias, cryptorchidism	Male	-	T	OT	+	+	71	Normal	Low	Low	NA	Tangshewinsirikul et al. [17], 2020
3	15	Scrotal mass	Phenotypic male	Male	+	OT	O	+	NA	87	Low	Normal	Normal	NA	Chouhan et al. [16], 2017
4	1.7	Atypical genitalia	NA	Male	-	OT	T	+	+	NA	NA	NA	NA	NA	Mao et al. [15], 2017
5	12	Female body habitus	Cryptorchidism	Male	+	O	T	-	+	88	Low	Normal	Low	No	Mohd et al. [8], 2016
6	16	Cyclic hematuria	Cryptorchidism	Male	+	T	O	+	NA	80	Low	Normal	Normal	No	Talreja et al. [9], 2015
7	0.42	Atypical genitalia	Hypospadias, cryptorchidism, micropenis	Male	NA	T	O	+	NA	66^R^ 84^L^	Normal	Normal	Normal	NA	Ozsu et al. [10], 2013
8	13	Left scrotal pain	Cryptorchidism	Male	+	T	O	+	+	70	NA	NA	NA	No	Kanaka et al. [11], 2007
9	14.5	Left scrotal pain, cyclic hematuria	Hypospadias	Male	+	T	OT	+	NA	85	Low	High	High	Yes	Isguven et al. [14], 2005
10	16	Atypical genitalia	Bifid scrotum hypospadias, cryptorchidism	Male	NA	OT	O	+	-	72	Low	High	High	No	Torres et al. [13], 1996
11	14	Left scrotal pain	Hypospadias, cryptorchidism	Male	+	OT	OT	NA ^a^	+	50	Low	Normal	High	No	Bergmann et al. [18], 1989
12	16	Bilateral gynecomastia	Unilateral cryptorchidism	Male	+	OT	O	+	-	72	Low	Normal	Normal	No	Pérez et al. [5], 1981
13	0.33	Atypical genitalia	Perineal hypospadias, cryptorchidism	Male	NA	T	O	+	+	62	NA	NA	NA	NA	Butler et al. [12], 1969

In our patient, a uterus (15 mm) with endometrium and a vaginal cavity were observed. Similar to most reported cases, the presence of Müllerian derivatives was confirmed in all but one case, which only had Wolffian ducts [8]. This latter was present in 41.6% (5/12) of cases with a uterus [11,12,15,17,18]. The coexistence of Wolffian and Müllerian derivatives in the same patient with an ovary on one side and an ovotestis on the other has not been reported.

In patients with mosaic 46,XX/47,XXY and OT-DSD, the most common gonadal combinations are pure ovary and pure testis (38.5%, 5/13), followed by ovotestis and ovary (30.8%, 4/13), and ovotestis with testis (23%, 3/13). Bilateral ovotestis is the rarest combination, found in only one case (7.7%) (Table 2). Consistent with common OT-DSD patterns [7], among the 26 gonads identified here, the most frequent types were ovary and ovotestis, each found in nine of 26 gonads (34.6% each) [7-18]. This was followed by testis, found in eight of 26 gonads (30.7%) [8-12,14,15,17]. The ovary was often located on the left side (7/13 cases), while the testis was more frequently found on the right side (6/13 cases). In contrast, the ovotestis did not show any specific pattern in body orientation.

**Figure 4:**
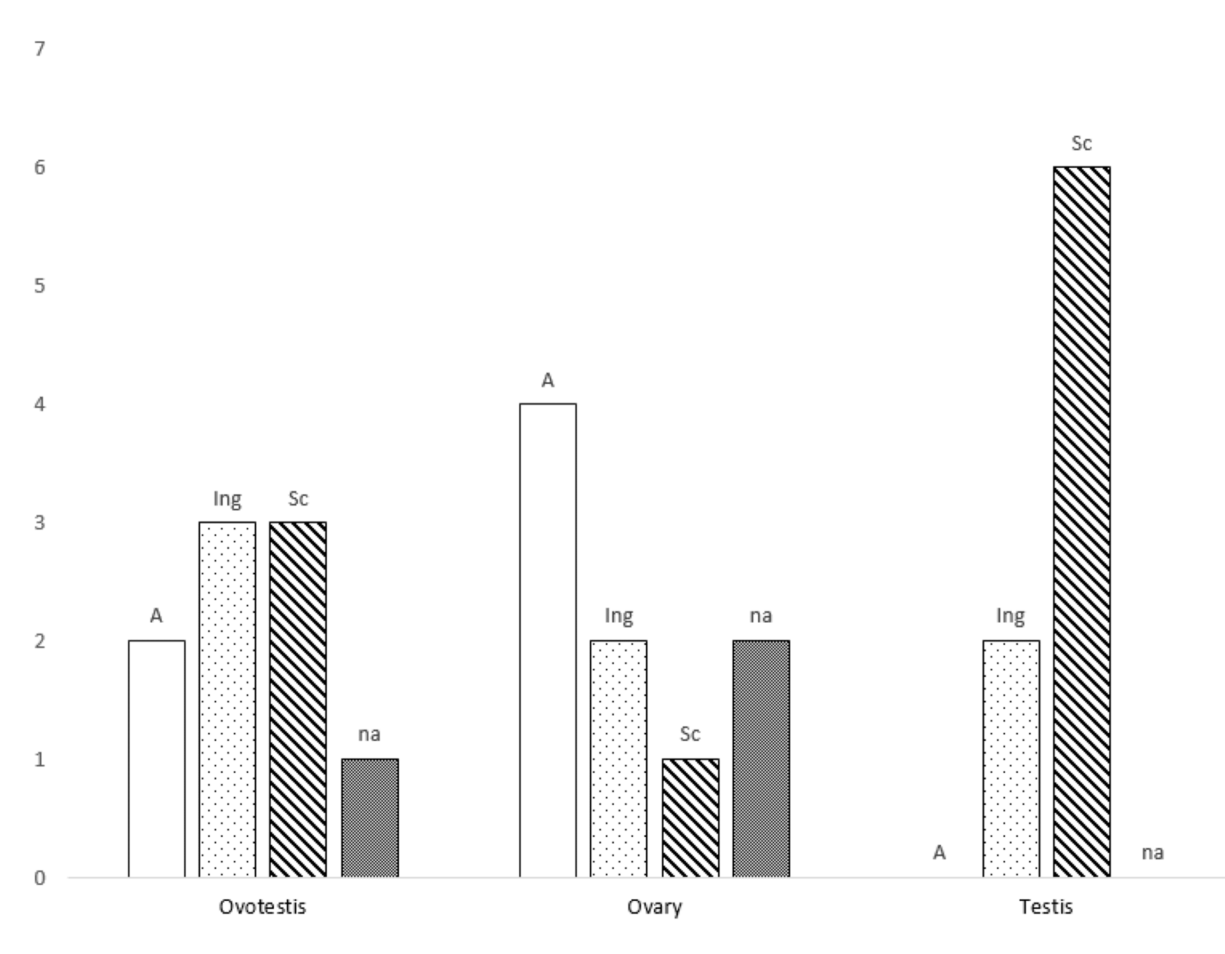
The anatomical location and types of gonads in 13 cases of 46,XX/47,XXY mosaicism. A: abdomen; Ing: inguinal canal; Sc: scrotal; na: data not available.

Our 46,XX/47,XXY patient is unique as the ovarian structure was located on the right side of the abdomen, while the left side contained an ovotestis. This differs from all previously reported cases of 46,XX/47,XXY with both an ovary and an ovotestis [5,13,16]. Moreover, gonads can be found in various positions, including labioscrotal folds, inguinal regions, or the pelvic/abdominal area. When data were available, the ovary was often located in the abdomen rather than the inguinal or labioscrotal folds, while the ovotestis could be found in all positions without a specific pattern (Figure 4). The position of the gonads primarily seems to depend on the amount of testicular tissue present: it is more likely the gonads descend with a greater amount of testicular tissue [7].

The presence of the Y chromosome, along with the genes involved in gonadal development, plays a crucial role in normal testicular development, contributing to the regression of Müllerian derivatives through the secretion and action of anti-Müllerian hormone and facilitating the formation of Wolffian derivatives through the local action of testosterone [22,23]. In mosaic cases, gonadal differentiation toward either pure testes or pure ovaries depends on the predominant cell line within the gonads [7]. Our patient's molecular analysis, which revealed no mutations in the related genes, supports this explanation. Notably, a reported case of sex discordance in monozygotic 46,XX/47,XXY twins revealed a higher percentage of 46,XX cell lines in all examined tissues, including peripheral blood lymphocytes, buccal smears, and urinary sediments, of the phenotypic girl twin compared to the male twin [24].

In our patient's peripheral blood, the karyotype showed a predominance of 46,XX cells (54%) over 47,XXY cells (46%). This predominance of 46,XX cell lines in peripheral blood has been observed in all reported cases here, regardless of the degree of genital appearance or gonadal differentiation (Table 2). Additionally, there was no correlation between the percentage of Y-bearing chromosome cells in this mosaicism and the degree of masculine appearance observed. The origin of the extra X chromosome is rarely determined; only two cases in the literature have described its origin, and both confirmed a paternal origin [17,25].

In our patient, the high levels of AMH, combined with the persistence of Müllerian ducts and the absence of Wolffian ducts, were intriguing. While the slightly elevated AMH and normal FSH may indicate normal Sertoli cell function [26], the persistence of Müllerian structures might suggest inadequate effectiveness in inducing Müllerian duct regression. The slightly elevated LH and T levels indicate that Leydig cells are somewhat activated but insufficient for complete sexual differentiation. Therefore, the persistence of Müllerian ducts alongside the absence of Wolffian ducts suggests that the hormonal signals for masculinization (testosterone) and Müllerian duct regression (AMH) are not functioning, as they should.

A second hormonal measurement at one month of age revealed that the initially high AMH levels at birth had normalized, indicating a possible typical postnatal adjustment of Sertoli cell function. This change might also suggest that the ovary contributed to the initial higher AMH levels or that there were a higher number of primordial follicles. Nevertheless, the persistently slightly elevated LH and T levels suggest ongoing Leydig cell activity and possible hormonal imbalances. Further exploration of gonadal function could provide insights for determining the most effective therapeutic outcomes. This combination of findings highlights the complex nature of OT-DSD, where hormone levels alone do not fully explain the presence of certain developmental features. Overall, in most cases where data were available, testosterone levels were low, FSH was normal in the prepubertal stage and high during puberty, while LH levels were variable (Table 2).

Furthermore, fertility is reduced in male individuals with OT-DSD or KS [7,27]. However, spermatogenesis has been observed in only two OT-DSD individuals [7]. Whereas, 21 pregnancies occurred in 10 females with OT-DSD, two of these later developed tumors in their ovotestis. However, none of these females had 46,XX/47,XXY mosaicism [7]. Interestingly, paternity might be possible through assisted reproductive techniques like intracytoplasmic sperm injection (ICSI) in both mosaic and non-mosaic KS men [28]. Nevertheless, there is an elevated incidence of chromosomal aneuploidies in the offspring [29]. To date, there have been no reports of successful reproduction in OT-DSD with 46,XX/47,XXY mosaicism.

Overall, compared to other types of DSDs, OT-DSD patients have a lower risk of germ-cell tumors [7,30]. None of the 12 cases reported here documented tumors. However, in our patient, vigilance for potential malignant development with age is recommended, requiring a long-term follow-up. Previous literature has documented that individuals with KS (47,XXY) often experience language disorders and reading disabilities [31]. However, these issues are less common in individuals with mosaicism 46,XX/47,XXY [31]. Only one patient in this review presented learning difficulties [14].

## Conclusions

The presence of atypical genitalia represents a challenge in sex assignment and leads to unpredictable clinical outcomes. It is important to recommend a cytogenetic analysis for all patients with cryptorchidism to detect any hidden mosaicism. Consistent observation of persistent Müllerian structures is a significant finding that may guide management decisions for such cases. The external genitalia predominantly presented a nearly male phenotype, thus influencing the parents' preference for raising their children as male. Furthermore, it is essential to consider fertility potential and genital alignment when deciding on the sex of rearing. No gonadal tumors were documented in the reported cases. Finally, a multidisciplinary management approach is needed, involving geneticists, endocrinologists, surgeons, and psychiatrists to ensure comprehensive care and optimal outcomes for these patients.

## Appendices

**Table 3:** List of genes involved in sexual differentiation disorders included in the next-generation sequencing panel. OMIM: Online Mendelian Inheritance in Man.

Type, Name	OMIM
GENE_CDS,STAR	600617
GENE_CDS,WT1	607102
GENE_CDS,GATA4	600576
GENE_CDS,DHCR7	602858
GENE_CDS,HSD17B3	605573
GENE_CDS,WNT5A	164975
GENE_CDS,CYP17A1	609300
GENE_CDS,CYP11B1	610613
GENE_CDS,HSD3B2	613890
GENE_CDS,INSL3	146738
GENE_CDS,AMHR2	600956
GENE_CDS,DHH	605423
GENE_CDS,LHCGR	152790
GENE_CDS,NR5A1	184757
GENE_CDS,AKR1C2	600450
GENE_CDS,MAMLD1	300120
GENE_CDS,CBX2	602770
GENE_CDS,NR0B1	300473
GENE_CDS,CYP11B2	124080
GENE_CDS,CYP19A1	107910
GENE_CDS,FGFR1	136350
GENE_CDS,SOX9	608160
GENE_CDS,CHD7	608892
GENE_CDS,RSPO1	609595
GENE_CDS,AKR1C1	600449
GENE_CDS,HSD17B1	109684
GENE_CDS,CYP11A1	118485
GENE_CDS,SRD5A1	184753
GENE_CDS,AKR1C3	603966
GENE_CDS,POR	124015
GENE_CDS,SULT2A1	125263
GENE_CDS,HSD3B1	109715
GENE_CDS,NR3C1	138040
GENE_CDS,DMRT1	602424
GENE_CDS,HSD11B1	600713
GENE_CDS,SOX10	602229
GENE_CDS,CHD4	603277
GENE_CDS,MAP3K1	600982
GENE_CDS,ATRX	300504
GENE_CDS,CYB5A	613218
GENE_CDS,FGFR3	134934
GENE_CDS,AMH	600957
GENE_CDS,ANOS1	300836
GENE_CDS,DHX37	ENSG00000150990
GENE_CDS,DMRT2	604935
GENE_CDS,SOX3	313430
GENE_CDS,TSPYL1	604714
GENE_CDS,WNT4	603490
GENE_CDS,AKR1C4	600451
